# Development of artificial intelligence edge computing based wearable device for fall detection and prevention of elderly people

**DOI:** 10.1016/j.heliyon.2024.e28688

**Published:** 2024-04-09

**Authors:** Paramasivam A, Ferlin Deva Shahila D, Jenath M, Sivakumaran T.S, Sakthivel Sankaran, Pavan Sai Kiran Reddy Pittu, Vijayalakshmi S

**Affiliations:** a Department of Biomedical Engineering, Vel Tech Rangarajan Dr. Sagunthala R&D Institute of Science and Technology, Chennai, 600062, India; b Department of Electronics and Communication Engineering, Vel Tech Rangarajan Dr. Sagunthala R&D Institute of Science and Technology, Chennai, 600062, India; c Department of Electronics and Communication Engineering, SRM Institute of Science and Technology, Kattankulathur, Chennai, 603203, India; d Department of Electrical and Computer Science Engineering, Bule Hora University, Oromia, Ethiopia; e Department of Biomedical Engineering, Kalasalingam Academy of Research and Education, Krishnankoil, Virudhunagar, 626126, India

**Keywords:** Accelerometer sensor, Deep learning models, Fall detection, Internet of things, Secure pairing, Wearables

## Abstract

Elderly falls are a major concerning threat resulting in over 1.5–2 million elderly people experiencing severe injuries and 1 million deaths yearly. Falls experienced by Elderly people may lead to a long‐term negative impact on their physical and psychological health conditions. Major healthcare research had focused on this lately to detect and prevent the fall. In this work, an Artificial Intelligence (AI) edge computing based wearable device is designed and developed for detection and prevention of fall of elderly people. Further, the various deep learning algorithms such as Convolutional Neural Network (CNN), Recurrent Neural Network (RNN), Long Short-Term Memory (LSTM), Gated Recurrent Unit (GRU) are utilized for activity recognition of elderly. Also, the CNN-LSTM, RNN-LSTM and GRU-LSTM with and without attention layer respectively are utilized and the performance metrics are analyzed to find the best deep learning model. Furthermore, the three different hardware boards such as Jetson Nano developer board, Raspberry PI 3 and 4 are utilized as an AI edge computing device and the best deep learning model is implemented and the computation time is evaluated. Results demonstrate that the CNN-LSTM with attention layer exhibits the accuracy, recall, precision and F1_Score of 97%, 98%, 98% and 0.98 respectively which is better when compared to other deep learning models. Also, the computation time of NVIDIA Jetson Nano is less when compared to other edge computing devices. This work appears to be of high societal relevance since the proposed wearable device can be used to monitor the activity of elderly and prevents the elderly falls which improve the quality of life of elderly people.

## 1 Introduction

In India, nearly 1.5–2 million elderly people experience injuries every year due to falls, and 1 million lose their lives due to falls [1]. Falls are accidental events and the fall is caused due to loss of Center of Gravity (CoG) and no effort is made to restore balance or this effort is ineffective. Further, the falls are mostly caused due to unbalance, posture, vision impairment, foot problems, muscle weakness, chronic health conditions, depression, anxiety etc. and these falls would lead to a long‐term negative impact on the physical, psychological health and socioeconomic condition of the individual including fractures, joint dislocation, loss of independence, head trauma and tissue injuries [2]. All these would have a great impact on the quality of life [1,3,4]. Fear of falling creates negative effects and it can minimize/restricts the patient's activities, social interactions, and ultimately results in depression [5,6]. The risk of falling and fall related problems rises with age. However, many falls can be alerted and prevented with the help of technological advancements [[7], [8], [9], [10], [11], [12], [13], [14]].

Recent years, a lot of research has been undertaken on fall related systems. These fall related systems can be divided into two types based on data acquisition: context-aware and wearable systems [15]. Context-aware systems include installing devices in the different places where the user is being monitored. Wearable sensor-based systems provide real-time monitoring with the data acquired from the user. Wearable sensor-based systems seem to be becoming more common since they are highly precise at detecting falls despite the patient's environment (i.e., outdoors or indoors). These simple and low-power devices commonly consist of microcontrollers and inertial measurement sensors [16]. The inertial measurement sensors usually collects data which includes rotation, motion direction, and acceleration [17].

Fall-related systems are also divided into two research tracks, i.e., fall detection and fall prevention [18]. Fall detection helps the people to detect accurately whenever a fall occurs and notifies the fall incident quickly, so that time to reach and rescue the elderly can be reduced [19]. On the other hand, fall prevention helps the people to predict the fall through gait analysis which looks at how the person walk and helps to analyse even, uneven, climbing, and descending gait patterns. As walking on an even surface acts as a baseline, allowing deviations to be easily detected as the surface is smooth and consistent, the steps placed are evenly spaced and symmetrical, walking on uneven surfaces helps to detect any balance and helps to analyse the step length, stride, and foot placement. Furthermore, the climbing and descending stairs test balance, strength, coordination and help to analyse dorsiflexion and plantarflexion of the knee gives data about the swing phase and push off phase while walking. By combining this data with wearable technology and smart learning models makes it more dependable in predicting and correcting falls and posture recognition to recognize people with a higher risk of falling [[20], [21], [22], [23]].

Wearable-based fall systems combined with a deep learning model can be a more reliable technique to predict fall prevention, detection and posture correction [[14], [15], [16], [17], [18], [19]]. But, deep learning algorithms require high computational power for performing large operations. Running such deep learning models on a low-powered microcontroller board can lead to high-power consumption and long response times [24]. To meet the computational demands of deep learning models, the integration of edge computing devices gives a promising solution. With the help of edge computing, the wearable sensor-based fall detection can achieve enhanced efficiency and responsiveness [[25], [26], [27], [28], [29], [30]]. These devices can pre-process sensor data, extract relevant features, and even execute lightweight versions of deep learning models locally in the wearable sensors or at a nearby server. This real-time data processing at the edge not only reduces power consumption but also significantly shortens response times. Alongside faster response times, edge computing improves the privacy and security of fall detection systems. It prevents the need for sensitive sensor data to be sent across highly unsafe networks by processing it locally. This strategy helps to protect the individuals’ personal information.

The objective of this work is to design and develop an Artificial Intelligence Edge Computing based wearable device for fall detection of Elderly People using deep learning techniques.

## 2 Literature survey

In recent years, the aging population has grown significantly, presenting challenges for healthcare systems. Biswas et al. (2022) have presented a review to synthesize the evidence on the risk factors for falls among elderly people in India Also, the authors have discussed about the various risk factors such as geographic, environmental, lifestyle etc. Hussain et al. (2019), Cumming et al. (2000) and Joseph et al. (2019) have discussed the major issue of fatal injuries and hospitalizations resulting from accidental falls in the elderly population and how these falls are negatively impacting their lifestyle.

Several researchers have discussed about the Activity of Daily Life (ADL) and its recognition using modern techniques. Salah et al. (2022) & Usmani et al. (2021) have discussed about the context-aware systems and wearable sensor based systems developed for fall detection and the challenges and future trends. Chaccour et al. (2016) have discussed regarding the research tracks in fall related system. Xu et al. (2018), Kharb et al. (2011), Young et al. (2012) and Rupasinghe et al. (2019) have discussed about fall detection, fall prevention, walking patterns etc. Queralta et al.(2019), Chhetri et al. (2021), Yu et al. (2023), Wang et al. (2020), Salah et al. (2020) have designed, developed and discussed the different types of Real-time fall detection systems. Also, the authors have discussed about the wearable monitoring framework that predicts, safeguards, and notifies during and after a fall and also usage a deep neural network architecture combining Convolutional Neural Networks and Recurrent Neural Network for analysis of data acquired from the elderly. Chang et al. (2021), Al Nahian et al. (2021), Salah et al. (2022) and Kharb et al. (2011) have designed and discussed about pose detection and gait analysis which is helpful to estimate the activity of the person based on accelerometer readings and provide more efficient results.

Svoboda et al. (2022) conducted a study on running deep learning models on microcontrollers, the authors have discussed about the potential of microcontrollers (MCUs) and challenges faced while deploying Deep Neural Networks on MCUs with limited on-chip RAM (often <512 KB) and limited computing capabilities. Furthermore, the authors have delved into the ways of achieving fast DNN on MCUs. Also, the authors have formulated the practical guidelines to simplify the future deployment of DNN-based applications on microcontrollers. Chang et al. (2021), Lin et al. (2022), Sanchez et al. (2020) and Vimal et al. (2020) have designed and developed a fall detection and prevention devices which are deployed on to edge computing devices and discussed about the usage of edge computing to replace traditional computing methods for wearable sensor based systems. Lin et al. (2023) authors proposed an edge computing based device which detects bedside falls and recognizes the sleeping posture.

## 3 Proposed methodology

**Fig. 1:**
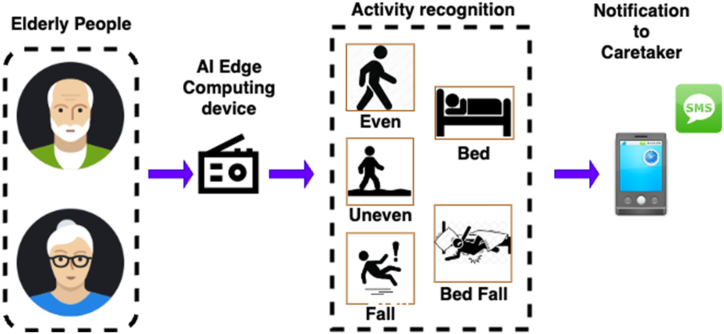
Overall Block diagram of proposed wearable device.

In this work, a wearable device for fall detection, prevention and posture correction of Elderly People is proposed. Fig. 1 shows the block diagram of proposed wearable device for detection of fall of elderly.

The proposed device is an Artificial Intelligence Edge Computing based wearable device which alerts the care takers of elderly people about their ADL including fall. Also, the proposed device is capable of identifying the abnormal postures of elderly and alerting the caretakers. The proposed wearable device can be fixed to any elderly people and the elderly can be monitored all day. Also, the caretakers can be notified through Short Messaging Service (SMS) about the abnormal events of the elderly since the edge computing wearable device is connected to internet. The ADL of elderly is updated continuously to Internet of Things (IoT) cloud platform and the activity of elderly can be monitored remotely.

### 3.1 Proposed fall detection and prevention framework

**Fig. 2:**
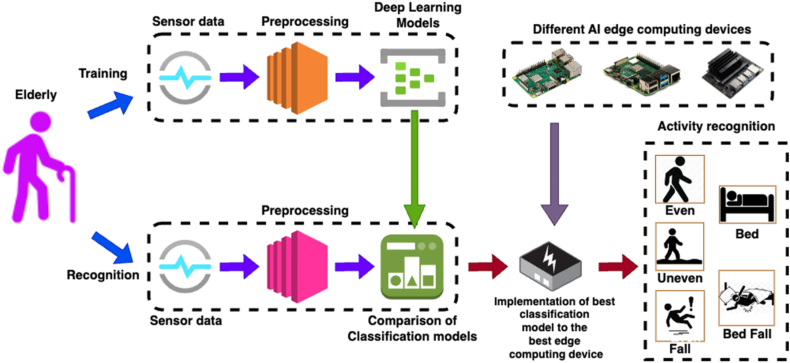
Block diagram of proposed fall detection approach.

Fig. 2 shows the block diagram of proposed approach for fall detection and prevention. Also, the proposed approach involves various components such as event sensor, data preprocessing, deep learning models, AI edge computing hardware and Graphical User Interface (GUI) for care takers.

#### 3.1.1 Event sensor

In this work, a triple axis accelerometer module (MPU6050) is utilized as an event sensor to measure x, y and z-axis of elderly people while performing various events such as fall, fall from bed, lying in bed, walking in even and uneven surface. Further, the MPU6050 module is cheap, compact and can be easily interfaced with any edge computing devices using Inter-Integrated Circuits (I2C) protocol. Also, it operates on input voltage ranges from 2.3 V to 3.4 V and has a sensitivity up to 131 LSBs/dps. The 3-axis sensor data is measured with respect to the activity of elderly people and is transmitted to the edge computing device.

#### 3.1.2 Data preprocessing

The data from the accelerometer sensor is stored in.csv files according to various events. This Input data is read in 15 separate files Even_x, Even_y, Even_z, Uneven_x, Uneven_y, Uneven_z, Fall_x, Fall_y, Fall_z, Bedfall_x, Bedfall_y, BedFall_z, Bed_x, Bed_y, Bed_z. This data is then further divided into sublists of length 80% for training and 20% for testing. Also, these data files are read in stratified order so that the data won't be overfitted during the training process. The signals acquired from the 3-axis sensor module consists of multiple unwanted frequency signals otherwise called as noises along with the ADL data. Further, the frequency components present in the ADL are analyzed using Fast-Fourier Transform (FFT). FFT is used to convert time domain signals to frequency domain which provides clarity about the frequency content in the signal. Since, the data from the 3-axis sensor is a time varying frequency signal, by observing the FFT plots gives no information about the time dependent frequencies i.e how frequency content varies with respect to the time. This information can be obtained by the spectral analysis. Additionally, the larger the window size used for FFT, the better frequency resolution will be made, but the time localization will be poor. Similarly, the smaller the window size, the better localization time, but frequency decomposition will be poor. So, window length of 128 was used with hamming window, as it gives the best frequency-time response for all the signals. To predict the human activity for every 2 s, training and testing data was segmented in the sequences of length 192 and each sequence was labeled with the correct label of activity. It is observed that there were no higher magnitude signals beyond 8Hz ADL of elderly and the signal with 0Hz frequency introduces DC components in the desired signal. So, the Finite Impulse Response (FIR) filter is designed to remove unwanted frequency components present in the acquired ADL signals. Also, the lower and the upper cut-off frequency is set to 4Hz and 8Hz respectively. In the preprocessing stage, the frequency components beyond this range are eliminated in the acquired ADL signals. Furthermore, the preprocessed signals are given to the various learning algorithms for activity recognition of the elderly.

#### 3.1.3 Deep learning models

Recent years, researchers have developed modern system models that can do classification/prediction from input datasets such as signals, images etc. called as Deep Learning. While performing detailed literature study and from study [31], it is evident that the LSTM is a good choice for fall detection systems since it has better performance. Also, the LSTM combined with RNN, CNN, GRU has better performance than individual models’ performance. So, the different deep learning-based techniques such as CNN, RNN, LSTM, GRU, CNN-LSTM, RNN-LSTM and GRU-LSTM are utilized in this work for fall detection and prevention of elderly. Also, the attention layer is added to three different deep learning-based models namely CNN-LSTM, RNN-LSTM and GRU-LSTM and the performance is analyzed.

A. **Convolutional Neural Network (CNN):** The proposed wearable device process accelerometer data to distinguish between various activities, including walking, running, ascending stairs and other forms of movement. This differentiation relies on the representation of sequential data points, and it's where Convolutional Neural Networks (1D CNNs) come into play, serving a crucial role in feature identification [28].

**Fig. 3:**
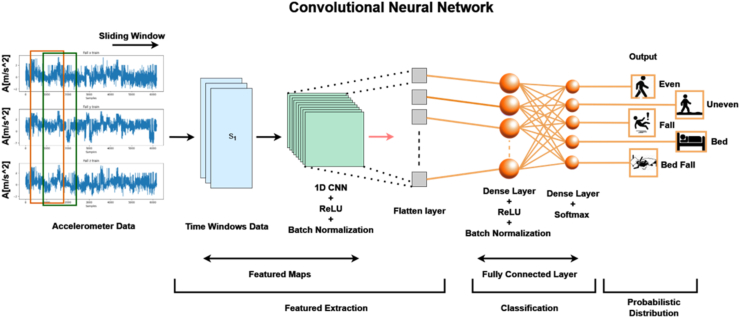
Architecture of proposed CNN model.

An architecture of proposed CNN model is shown in Fig. 3. Convolutional Neural Network (CNN) model, starts with a one-dimensional convolutional layer (Conv1D) with 64 filters and a kernel size of 3 is to extract relevant features from input sequences. Next, max-pooling is applied with a pool size of 2 to down sample the feature maps, followed by batch normalization to enhance convergence and generalization. The flattened representation is then given into a densely connected layer with 64 neurons, introducing non-linearity to the learned features. Another batch normalization step is incorporated before the final dense layer, the model effectively captures hierarchical patterns and spatial dependencies within the input data. The utilization of convolutional neural networks provides a robust and simple framework for sequence classification tasks. CNN contains an input layer, which takes in the sequential accelerometer data points, hidden layers for processing, and an output layer that provides an idea regarding the walking activity based on the data provided. Also, the various tools namely pooling and normalization can be used to refine the features taken from the input data and this process can enhance the network's ability to identify and classify the various activates based on the patterns observed in data.

**Fig. 4:**
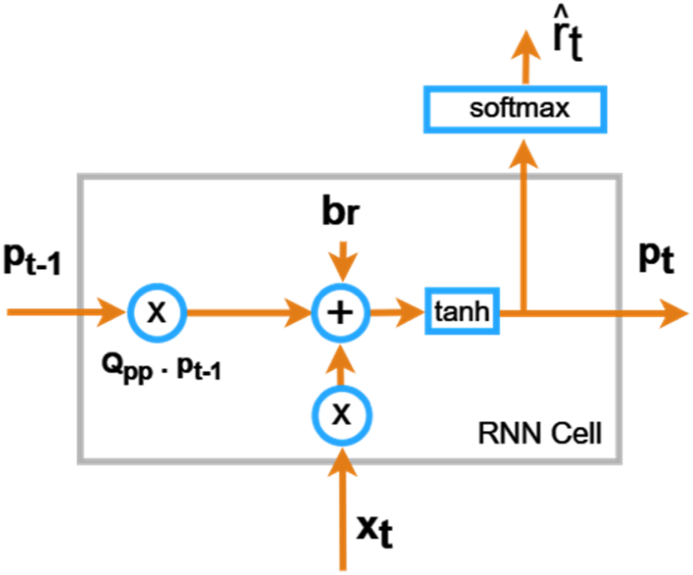
Basic block diagram of the proposed RNN.

B. **Recurrent Neural Network:** An artificial neural network especially Recurrent Neural Network (RNN) is designed to handle sequential data which shall be used for time-series prediction, language modelling etc. Also, the basic block diagram of the proposed RNN is shown in Fig. 4. This model starts with a convolutional layer and max-pooling to capture local features and simplify the input data it looks at patterns over time in the data. Batch normalization is used to make training more stable, leading to quicker learning and better handling of new data. The model ends with dense layers that summarize the learned features for efficient classification. This Simple RNN-based approach is good at finding short-term patterns.

RNN processes an input sequences element one at a time, maintaining a hidden state that captures information from previous inputs. In equation (1), at every time step t, the current input $x_{t}$ and the previous hidden state $p_{t-1}$ updates the hidden state $p_{t}$ which is given as [32]:

(1) $$p_{t}=\tanh\left(Q_{px}\cdot x_{t}+Q_{pp}\cdot p_{t-1}+b_{p}\right)$$

where,

$Q_{px}$ is the input weight matrix

$Q_{pp}$ is the hidden state weight matrix

$b_{p}$ is the bias term.

The non-linearity is introduced to the model using activation function tanh which allows to capture complex patterns. The hidden state at each time step acts as a memory that retains information from earlier inputs. The updated hidden state is then used for the next time step, creating a recurrent loop. At every time step t, the output is given as expressed by equation (2) [32]:

(2) $${\hat{r}}_{t}=soft\;\max\left(Q_{rp}\cdot p_{t}+b_{r}\right)$$

Here,

$Q_{rp}$ is the hidden state weight matrix connected to the output

$b_{r}$ is the output bias.

The output values is normalized using *SoftMax* function, turning them into probabilities. Also, the difference between predicted probabilities and the actual values are minimized using trained RNN [32].

C. **Long Short Term Memory:** The architecture of Long Short Term Memory **(**LSTM) utilizes the computation of the simple RNN. Further, it is comprised with a cell state in which the input cell memory is stored and converted to the output cell state. Long Short-Term Memory (LSTM) model starts with an input layer of one-dimensional time series data, the LSTM layer retain and utilizes long-term information by analysing the sequences. Using Batch normalization after the LSTM layer enhances training stability, facilitating faster convergence and improved generalization. The subsequent dense layers condense the learned features into a compact representation, with the final layer uses a SoftMax activation function for multi-class classification. The application of a batch normalization layer after both the LSTM layer and the first dense layer contributes to the model's overall robustness.

**Fig. 5:**
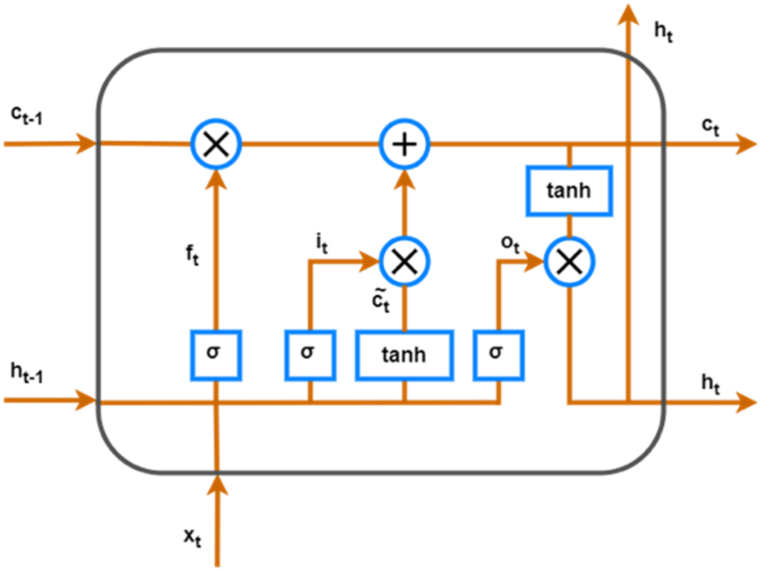
Basic block diagram for LSTM.

The architecture of the LSTM cell is shown in Fig. 5. Further, the LSTM cell consists of various subparts such as input gate & output gate, update gate and forget gate. Furthermore, the forget gate decides the data from previous memory units to be forgotten. Also, the nature of data information is decided by the input cell which shall be accepted into the neuron. The new long-term memory is produced using output gate and each cell is updated using update gate. Moreover, all these four components combinedly works together and interact in a specific way. The transfer of nature of information to the cell is determined by the input gate which is represented in Equation (3) [32]:

(3) $$i_{t}=\sigma \left(W_{i}*\left[h_{t-1},x_{t}\right]+b_{i}\right)$$

Forget gate determines the nature of information to be eliminated which is given by equation (4) [32]:

(4) $$f_{t}=\sigma \left(W_{f}*\left[h_{t-1},x_{t}\right]+b_{c}\right)$$

The cell state is update using update gate which is given by equations (5), (6)) [32]:

(5) $$c_{t}=\tanh\left(W_{c}*\left[h_{t-1},x_{t}\right]+b_{c}\right)$$

(6) $$c_{t}=f_{t}*c_{t-1}+i_{t}*c_{t}$$

The output gate is given by equations (7), (8)) [32],

(7) $$o_{t}=\sigma \left(W_{o}*\left[h_{t-1},x_{t}\right]+b_{o}\right)$$

(8) $$h_{t}=o_{t}*\tanh\left(c_{t}\right)$$

D. **Gated Recurrent Unit: The** Gated Recurrent Units (GRUs) is a simplified version of LSTM which is proposed by Chung et al. [33], GRU has advantages such as less training time, outstanding network performance.

**Fig. 6:**
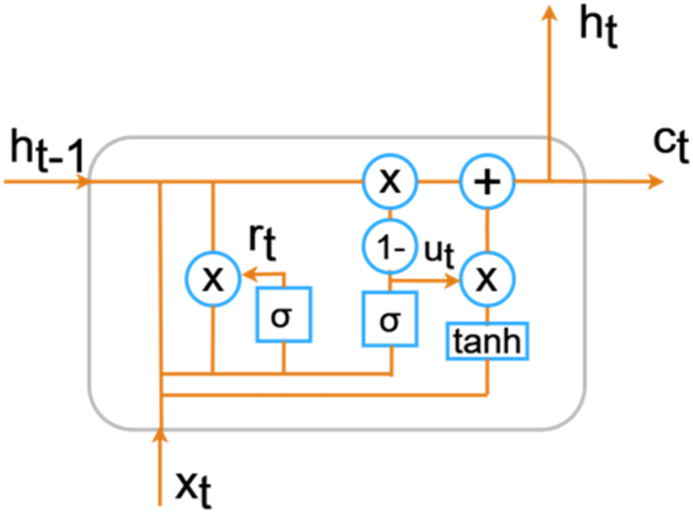
Basic block diagram of GRU.

The basic block diagram of GRU is shown in Fig. 6. The function of GRU and LSTM is similar however, the GRU utilizes one hidden state in which the input gate and the forget gate are merged together to form a single update gate. In GRU, the hidden and cell states are combined into one state. Due to this, the total number of GRU gates is reduced by half of the total number of LSTM gates which made GRU more popular. The update gate $u_{t}$ and reset gate $r_{t}$ are the two different gates of GRU. Also, the hidden state of the GRU is represented in equation (9) [33]:

(9) $$h_{t}=\left(1-u_{t}\right)*h_{t-1}+u_{t}*h_{t}$$

The update gate is represented in equation (10) and it determines number of the GRU unit has to be updated [33],

(10) $$u_{t}=\sigma \left(W_{u}*\left[h_{t-1},x_{t}\right]\right)$$

The reset gate is represented in equation (11) [33],

(11) $$r_{t}=\sigma \left(W_{r}*\left[h_{t-1},x_{t}\right]\right)$$

The hyperbolic tangent function of the reset gate is represented by equation (12) [33],

(12) $$h_{t}=\tanh\left(W*\left[r_{t}*h_{t-1},x_{t}\right]\right)$$

Gated Recurrent Unit (GRU) model starts with an input layer that accommodates a one-dimensional time series. The GRU layer captures temporal dependencies and patterns in the data. Batch normalization is applied to enhance stability during training, promoting faster convergence and improved generalization to unseen data. The subsequent dense layers help simplify the learned features into a condensed form. The final layer uses SoftMax to classify into multiple categories. Importantly, adding batch normalization after the GRU layer and the first dense layer makes the model more robust and speeds up training.

E. **Attention Layer: Attention** layers acts a filter and the purpose of these mechanisms is to let the model know that which part of the input data is essential during the training process, so that the model pays close attention to the relevant information, understands it and makes predictions easier in complex models. Further, the attention layer is like a smart way for computers to process information, inspired by how humans think. With this mechanism, models can focus on specific parts of the input and decide how important each part is. They highlight important areas in an image, capturing detailed features and making the model perform better. The application of attention layers proves particularly advantageous when dealing with 1D signals. This technology enhances the ability of models to find crucial patterns and minor details within a signal. By assigning varying degrees of importance to different segments of the signal, attention layers enable the model to concentrate on key features, such as significant peaks or critical transitions. This targeted approach facilitates more effective analysis and interpretation.
F. **CNN + LSTM:** Convolutional Neural Network (CNN) and Long Short-Term Memory (LSTM) Model starts with a one-dimensional convolutional layer to capture spatial features from the input sequence, employing convolutional filters to extract relevant patterns. Next, the Max Pooling layer down samples the spatial dimensions. The LSTM layer then processes the sequential data, capturing long-range dependencies and temporal patterns. We use batch normalization to make the training stable and quick. The flattened representation is then passed through densely connected layers for classification. This hybrid architecture leverages the strengths of both CNN and LSTM layers. It helps the model understand both spatial and temporal features, the model gets better at learning complex patterns and connections. This makes it perform better in tasks that need a good understanding of both spatial-temporal features.
G. **CNN + LSTM + ATT:** Convolutional Neural Network (CNN), Long Short-Term Memory (LSTM), and Attention mechanisms model begins with a 1D convolutional layer (conv1d) with 64 filters to capture spatial features from the input sequence followed by max pooling to capture hierarchical features in the input data. Then, an LSTM layer with 128 units is utilized to capture temporal features within the sequences. To enhance the model's stability and convergence, batch normalization is applied after both the convolutional and LSTM layers. Usage of attention mechanism, allows the model to dynamically focus on relevant parts of the input sequence during prediction, enhancing its ability to recognize and weigh important features. The attentive mechanism enables the model to assign varying levels of importance to different parts of the input sequence, optimizing its performance.
H. **GRU + LSTM:** Gated Recurrent Unit (GRU) and Long Short-Term Memory (LSTM) layers starts with a convolutional layer capturing local features from the data, followed by max-pooling to down sample the information. GRU and LSTM layers independently analyse the temporal dynamics of the data, extracting valuable patterns. The results from these layers are put together to make a complete representation. Batch normalization is used to make the learning process more stable, and the put-together features are flattened before going through dense layers for classification. This model uses the strong points of both GRU and LSTM architectures. GRU is good at catching short-term connections, while LSTM is great for identifying long-term patterns. This way, the model gets better at understanding complex data and can handle different types of information, especially in tasks like classification.
I. **GRU + LSTM + ATT:** Gated Recurrent Unit (GRU), Long Short-Term Memory (LSTM), and attention mechanism, the model takes input data sends it to a convolutional layer to capture local features. After that, a GRU layer and an LSTM layer independently analyse the temporal dynamics of the data, extracting valuable information from different aspects. The results from repeated layers are combined. A batch normalization layer helps keep things stable during training. An attention mechanism is added to highlight important parts of the sequence, helping the model focus on key segments. The last layers refine the features before giving a final classification. This integrated approach leverages the strengths of both GRU and LSTM architectures, along with attention mechanism, to make the model better at detecting patterns in data.
J. **RNN + LSTM:** Recurrent Neural Networks (RNN) and Long Short-Term Memory (LSTM) architectures starts with an LSTM layer, which captures temporal dependencies within the sequential data. To enhance stability during training and accelerate convergence, batch normalization is incorporated after the LSTM layer. A Simple RNN layer further extracts temporal features from the data. This combination of LSTM and Simple RNN allows the model to effectively capture both short-term and long-term patterns. The final dense layer utilizes a SoftMax activation function for multi-class classification. This approach improves the model ability to find complex temporal patterns.
K. **RNN + LSTM + ATT:** Recurrent Neural Networks (RNN) and Long Short-Term Memory (LSTM) with an attention mechanism. The model starts with an LSTM layer, capturing temporal dependencies within the sequential data. The incorporation of attention mechanism, implemented through a dot product operation, enables the model to focus on relevant segments of the input sequence, enhancing its sensitivity to critical patterns. Batch normalization is applied for stability during training, and a Simple RNN layer extracts additional temporal features. The final dense layer utilizes a SoftMax activation function for multi-class classification. This approach creates a model capable of discerning intricate patterns over varying time scales, improving the robustness of classification.

#### 3.1.4 AI edge computing hardware

In this work, the three different System on Chip (SoC) device such as Raspberry PI 3 Model B+, Raspberry PI 4 Model B and NVIDIA Jetson Nano developer board are utilized as an AI edge computing device since these less expensive, compact, reliable, easy to implement etc. Furthermore, the three different edge computing devices are compared with respect to cost, compactness, energy consumption and time for computation. The controllers except Raspberry PI 3 Model B + Controller utilized in this work have 4 GB RAM whereas the maximum RAM size of Raspberry PI 3 Model B+ is around 1 GB RAM. Also, the optimal device is identified and is utilized for the development of wearable based fall detection device of elderly.

#### 3.1.5 GUI for care taker

In this work, a webpage is designed and developed using Hyper Text Markup Language (HTML) code for the monitoring the ADL of elderly which can be accessed through smart phones or computer system. Further, the developed webpage is tethered using opensource tunnel.sh and the webpage can be accessed by the caretaker remotely to monitor the activity of the elderly people. The HTML code and deep learning code for activity recognition is uploaded to the edge computing devices. Also, the caretaker will get message once the elderly fall is occurred. However, the edge computing devices are connected to internet, the messaging service is obtained using Twilio service provider. The caretaker's mobile number is coded inside the edge computing device and once fall is occurred, the device requests the Twilio service to send Short Messaging Service (SMS) to caretaker and the caretaker will be notified.

### 3.2 Process of proposed fall detection method

**Fig. 7:**
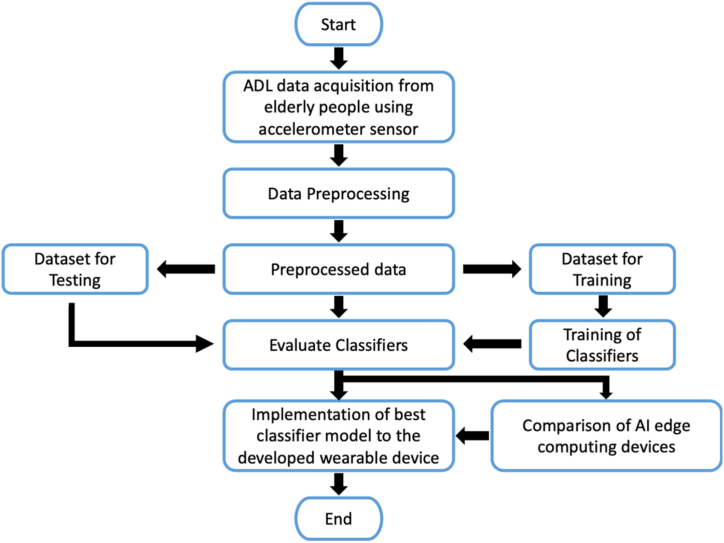
Flowchart for proposed fall detection approach.

Fig. 7 shows the flowchart for proposed approach for fall detection and prevention. The process flow of proposed fall detection and prevention approach consists of various stages such as ADL data acquisition and preprocessing, classifier training & testing, integration of classifier model to the developed wearable-based fall detection device and notification to caretakers.

The signals from the accelerometer sensor are preprocessed and the noises are removed. While observing the frequency values of three different axis namely x, y and z axis sensed by the accelerometer sensor, none of the signals have frequency beyond 8Hz during various event such as fall, fall from bed, lying in bed, walking in even and uneven surface. Also, the preprocessed sensor data is divided into two parts; one for training phase and another for testing phase. In this work, 80% of accelerometer sensor data is utilized for training the classifier models and 20% of accelerometer sensor data is utilized for testing the classifier models. The performance metrics of individual models namely CNN, RNN, LSTM, GRU and three different deep learning models namely CNN-LSTM, RNN-LSTM and GRU-LSTM with and without attention layer respectively are evaluated and compared. Also, the best deep learning model is identified and is implemented in three different edge computing devices. Furthermore, the efficacy of all the edge computing devices was analyzed and the best device was considered as an artificial intelligence edge computing based wearable device for fall detection and prevention of elderly people.

## 4 Experimentation, results and discussion

In this work, two different stages were performed namely the design & analysis of best classifier model and development of a wearable device for Fall Detection of elderly people. Furthermore, the design and analysis of the best classifier model involves the evaluation of various deep learning models such as CNN, RNN, LSTM, GRU and three different deep learning models namely CNN-LSTM, RNN-LSTM and GRU-LSTM with and without attention layer respectively with performance metrices. Also, the wearable device was designed and deep learning model with better performance metrics were deployed which acts as an edge device for fall detection and prevention of elderly people.

### 4.1 Design & analysis of classifier model

In this work, an accelerometer sensor signals were collected from elderly without any physical impairments ranging between 60 and 70 years old. Furthermore, the accelerometer sensor signals were collected from 120 individuals out of which 82 are male and 38 are female. Also, the informed consent was obtained and experimental procedures were clearly explained to the elderly people. The elderly people involved in this study were asked to perform various activities such as fall, fall from bed, lying in bed, walking in even and uneven surface and the accelerometer sensor signals were recorded. Also, the safe tests in a controlled setting were created. Older individuals were guided to pretend they were falling, but onto a soft bed to avoid any harm. This ensured their safety during the simulations. Also, the extra cushioning in different spots were added to catch the falls and reduce any possible risks. By performing such procedures, the useful and real information was gathered to improve detection about falls.

**Fig. 8:**
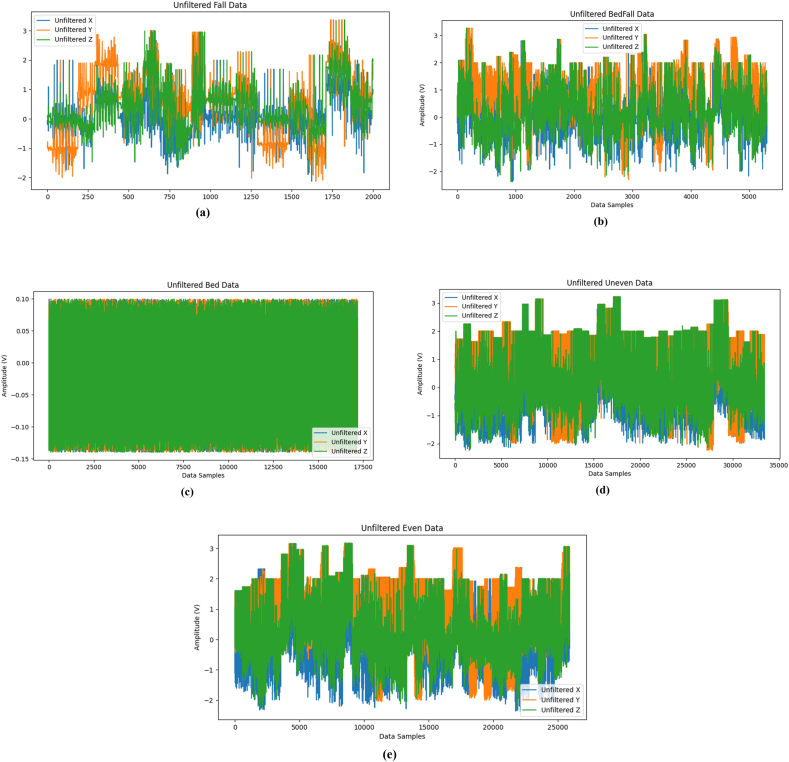
Unfiltered Accelerometer data collected from elderly during various events (a) fall, (b) fall from bed, (c) lying in bed, (d) walking in uneven surface and (e) walking in even surface.

**Fig. 9:**
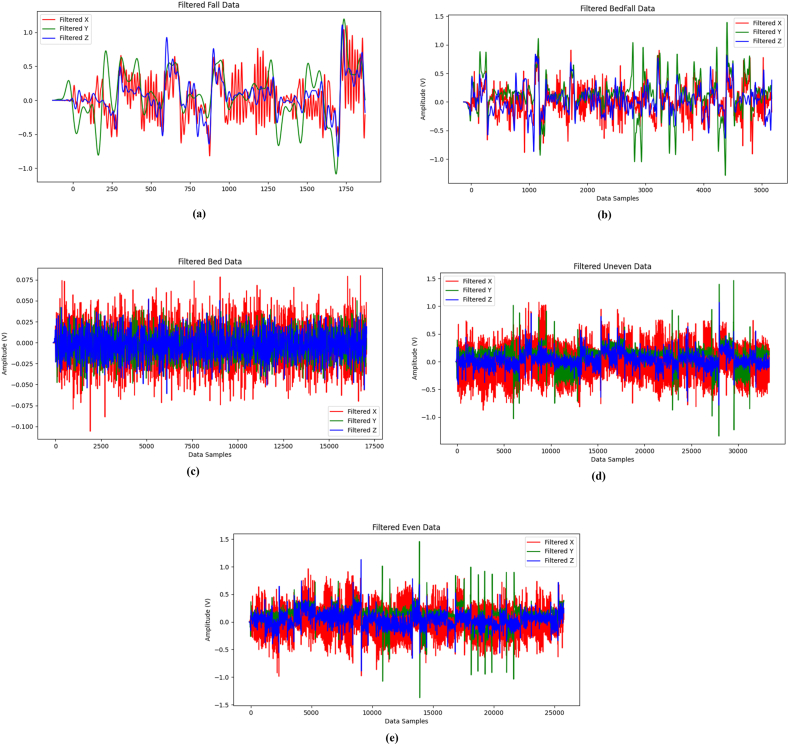
Filtered Accelerometer data collected from elderly during various events (a) fall, (b) fall from bed, (c) lying in bed, (d) walking in uneven surface and (e) walking in even surface.

Fig. 8 (a)-(e) show the unfiltered accelerometer data taken from elderly during various events such as fall, fall from bed, lying in bed, walking in uneven and even surface respectively. It is observed that the 3-axis namely x, y and z axis sensor signals during walking in even surface are plotted as amplitude versus data samples. The sensor signals were acquired with a sampling time of 0.05 Hz and the total of 1800 data points were recorded for 2 min. Also, these raw signals were filtered and the filtered accelerometer data taken from elderly during various events such as fall, fall from bed, lying in bed, walking in uneven and even surface respectively are shown in Fig. 9 (a)-(e). It is seen that the filtered accelerometer data has less noise when compared to unfiltered accelerometer data. Additionally, the filtering was done to eliminate the noises and to improve the performance of the utilized deep learning models.

**Fig. 10:**
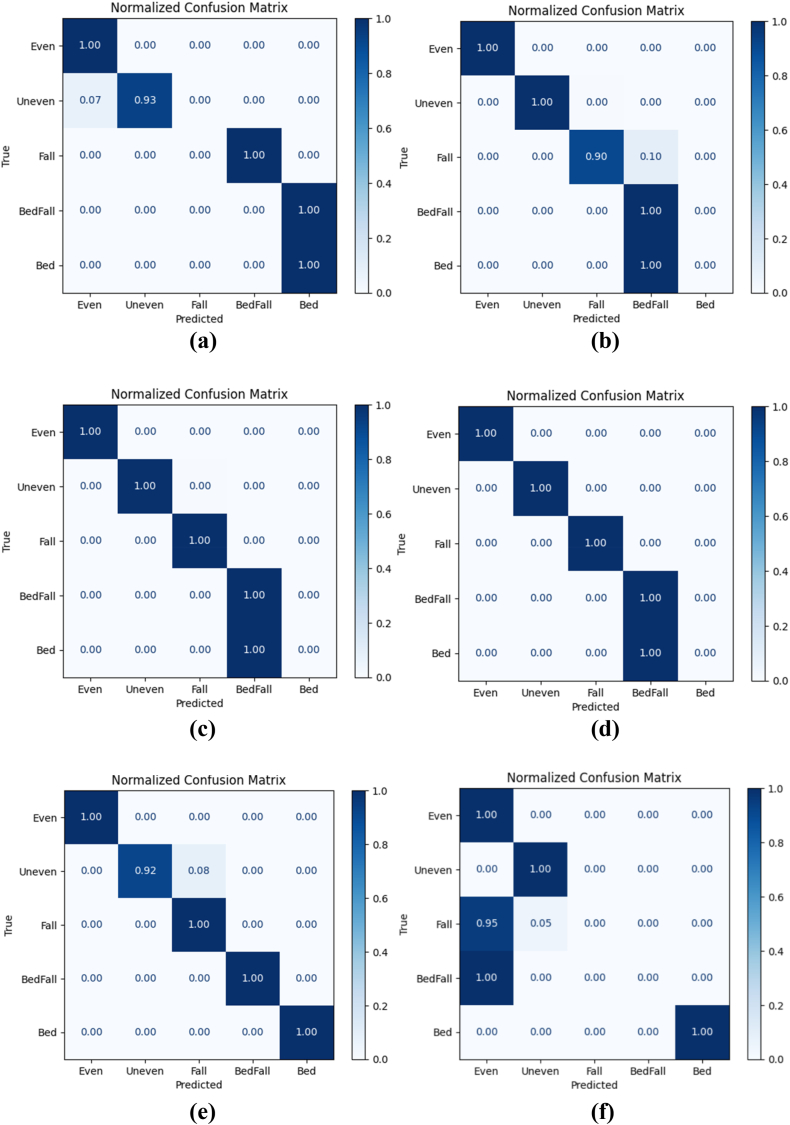

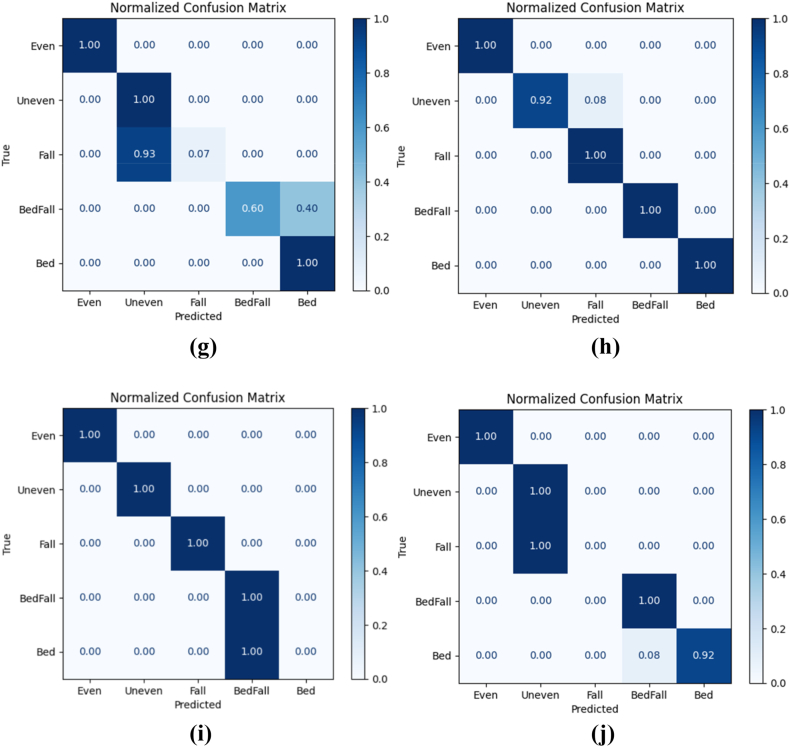
Confusion matrix of various deep learning models (a) RNN (b) CNN (c) GRU (d) LSTM (e) CNN-LSTM (f) RNN-LSTM (g) GRU-LSTM (h) CNN-LSTM with attention (i) RNN-LSTM with attention (j) GRU-LSTM with attention.

The filtered sensor signals for activities such as fall, fall from bed, lying in bed, walking in uneven surface of elderly were given to the utilized deep learning models. Also, the 80% of the collected sensor signals were given for training the models and 20 % of the collected sensor signals were given for testing the models in a stratified fashion. The normalized confusion matrix of various deep learning models such as RNN, CNN, GRU, LSTM, CNN-LSTM, RNN-LSTM, GRU-LSTM, CNN-LSTM with attention, RNN-LSTM with attention and GRU-LSTM with attention are shown in Fig. 10 (a) – (j). From these confusion matrixes, the performance metrics namely accuracy, recall, precision, F1_Score, model training time and testing time of various deep learning models such as RNN, CNN, GRU, LSTM, CNN-LSTM, RNN-LSTM, GRU-LSTM, CNN-LSTM with attention, RNN-LSTM with attention and GRU-LSTM with attention were evaluated.

#### 4.1.1 Evaluation metrics

In this work, the performance metrics such as accuracy, recall, precision, F1_Score, model training and testing time in seconds were computed to evaluate the performance of various deep learning models. Since, the proposed work has multiclass, the macro-averaged based metrics were used to evaluate the classification of deep learning classification models and it is given by.

A. **Accuracy:** The accuracy of the specific class of a given classifier is calculated as [34]:

(13) $$Accuracy=\frac{TP+TN}{TP+TN+FP+FN}$$

where, TP - True Positive.

TN - True Negative.

FP - False Positive.

FN - False Negative.

Once the accuracy is calculated for each class, the accuracies across all classes is averaged to find the macro-averaged accuracy. If there are N classes, then

(14) $${Accuracy}_{macro}=\frac{1}{N}\sum_{i=1}^{N}{Accuracy}_{i}$$

B. **Recall:** Recall is otherwise called as sensitivity which is calculated for the specific class of a given classifier as [34]:

(15) $$Recall=\frac{TP}{TP+FN}$$

Once the recall is calculated, the recalls across all classes is averaged to find the macro-averaged recall. If there are N classes, then

(16) $${Recall}_{macro}=\frac{1}{N}\sum_{i=1}^{N}{Recall}_{i}$$

C. **Precision:** Precision of the specific class of a given classifier is defined as follows [34],

(17) $$Precision=\frac{TP}{TP+FP}$$

Once the precision is calculated, the precision across all classes is averaged to find the macro-averaged precision. If there are N classes, then

(18) $${Precision}_{macro}=\frac{1}{N}\sum_{i=1}^{N}{Precision}_{i}$$

D. **F1_Score:** The harmonic mean of precision and recall for each class is defined as a F1_Score which is given by Ref. [34],

(19) $$F1_{Score}=\frac{2*precision*recall}{precision+recall}$$

Also, the macro-averaged F1_Score is utilized in this work to assess the overall performance of classifier model across all classes irrespective to imbalances in classes.

E. **Training and Testing Time:** The time of training and testing phase of individual different deep learning models and combined models such as CNN-LSTM, RNN-LSTM and GRU-LSTM with and without attention layer respectively are utilized in this work were computed. The network parameters namely the number of layers and units of the CNN-LSTM with attention layer is presented in Table 1.

**Table 1:** Network parameters namely number of layers and units.

CNN-LSTM with Attention Layer	Output Shape	Parameter
conv1d_91 (Conv1D)	(None, 254, 64)	256
max_pooling1d_91 (MaxPooling1D)	(None, 127, 64)	0
lstm_123 (LSTM)	(None, 127, 128)	98816
batch_normalization_234 (Batch Normalization)	(None, 127, 128)	512
flatten_91 (Flatten)	(None, 16256)	0
dense_194 (Dense)	(None, 64)	1040448
batch_normalization_235 (Batch Normalization)	(None, 64)	256
dense_195 (Dense)	(None, 5)	325
Dense Layer 6 (Dense):		
Number of units/neurons: 64		
Activation function: relu		
Input dimension: 16256		
Dense Layer 8 (Dense):		
Number of units/neurons: 5		
Activation function: softmax		
Input dimension: 64

The deep learning models were simulated using Google Co-laboratory in which high performance T4 Graphical Processing Unit (GPU) is used. Also, the models were tested on edge devices to provide a more realistic performance assessment. The.h5 model files generated during training were utilized for testing on these edge devices. This approach was chosen strategically, as Co-lab offered significant computational power for intensive training, while testing on edge devices resembled real-world usage scenarios. The value of performance metrics of various deep learning models is presented in Table 2.

**Table 2:** Performance metrics of various deep learning models.

Methods	Accuracy (%)	Recall (%)	Precision (%)	F1_Score	Training Time (Sec)	Testing Time (Sec)
RNN	83	59	53	0.55	65.64	1.83
CNN	80	78	64	0.67	4.70	0.07
GRU	81	80	65	0.68	65.11	0.58
LSTM	81	80	65	0.68	112.24	0.82
CNN-LSTM	90	71	70	0.67	68.46	0.85
RNN-LSTM	85	61	66	0.52	57.45	1.04
GRU-LSTM	89	73	95	0.75	1296.34	9.30
CNN-LSTM with attention	97	98	98	0.98	189.28	1.01
RNN-LSTM with attention	87	80	70	0.73	47.10	1.14
GRU-LSTM with attention	92	78	68	0.71	716.71	4.85

**Fig. 11:**
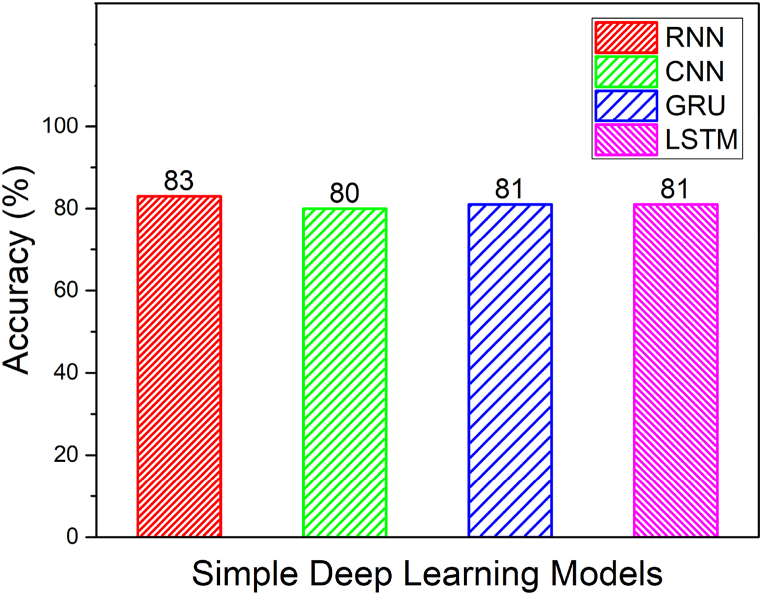
Accuracy Metrics of Simple Models (CNN vs. RNN vs. GRU vs. LSTM).

**Fig. 12:**
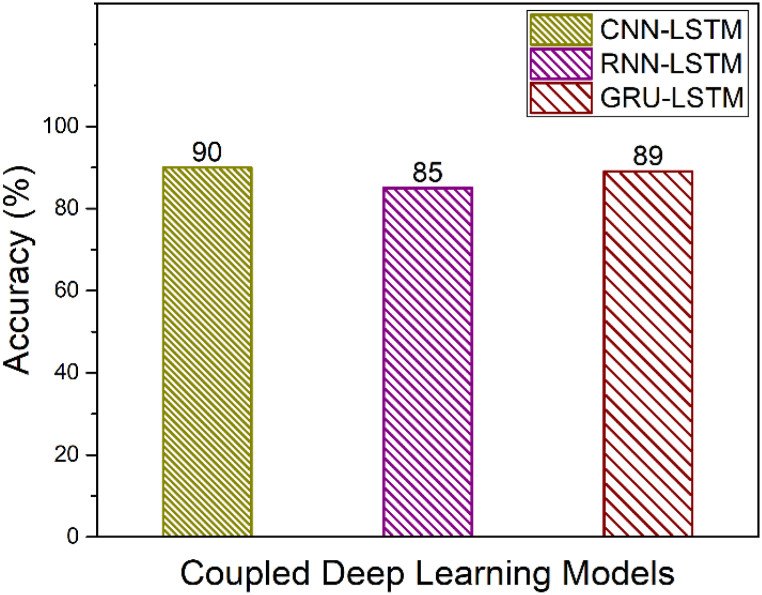
Accuracy Metrics of Coupled Models (CNN + LSTM vs. RNN + LSTM vs. GRU + LSTM).

**Fig. 13:**
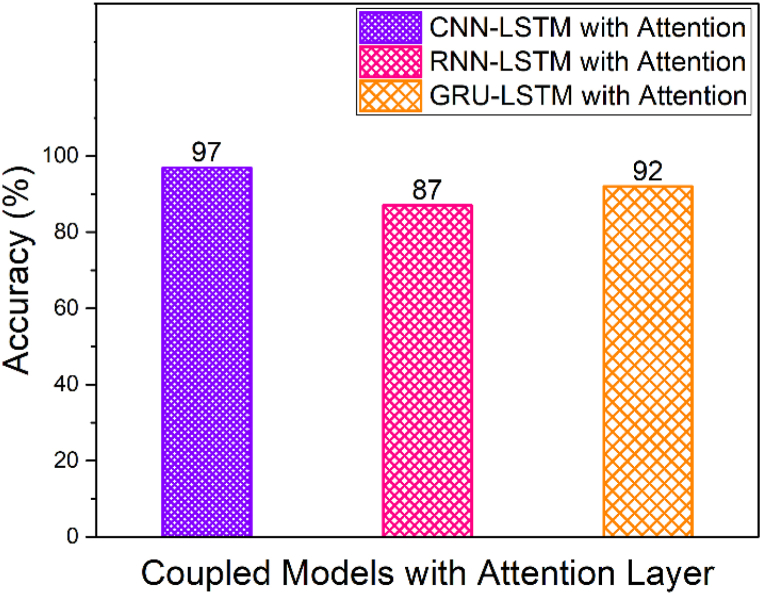
Accuracy Metrics of Coupled Models with Attention Layer (CNN + LSTM + ATT vs. RNN + LSTM + ATT vs. GRU + LSTM + ATT).

From Table 2 and it is seen that the accuracy of CNN-LSTM with attention layer is 97% which is higher when compared to the other deep learning models. Furthermore, the recall, precision and F1_Score of the CNN-LSTM with attention layer is 98, 98 and 0.98 respectively whereas the model training and testing time is 189.28 and 1.01 s respectively. Since, the training time of CNN-LSTM with attention is high when compared to CNN, RNN, LSTM, GRU, CNN-LSTM, RNN-LSTM and RNN-LSTM with attention, the training of the CNN-LSTM with attention was done high performance machine and the knowledge model was built h5 extension file. This h5 file directly uploaded to the edge computed device to avoid huge training time. Fig. 11 shows the macro averaged accuracy of simple models namely CNN, RNN, GRU and LSTM. Furthermore, the macro averaged accuracy of Coupled Models namely CNN + LSTM, RNN + LSTM and GRU + LSTM is shown in Fig. 12. Also, the macro averaged accuracy of Coupled Models with Attention layer namely CNN + LSTM + ATT, RNN + LSTM + ATT and GRU + LSTM + ATT is shown in Fig. 13.

**Fig. 14:**
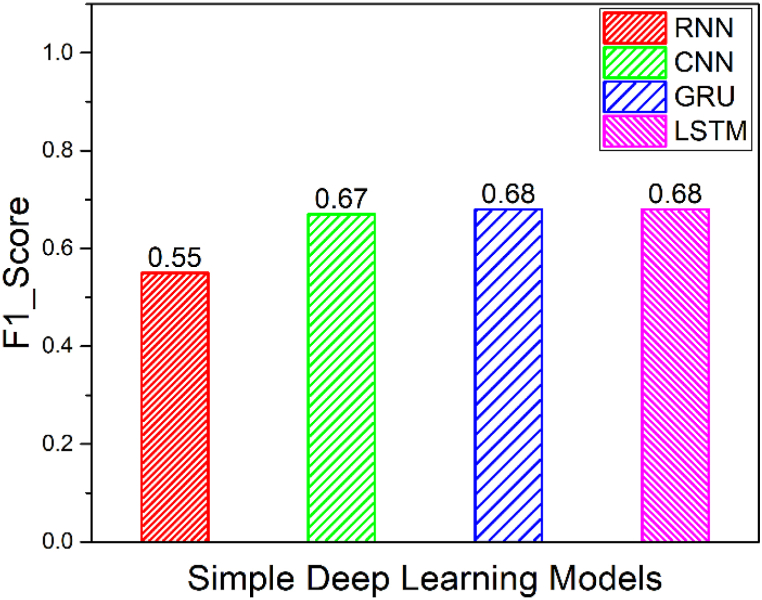
Comparison of F1_Scores of Simple Models (CNN vs. RNN vs. GRU vs. LSTM).

**Fig. 15:**
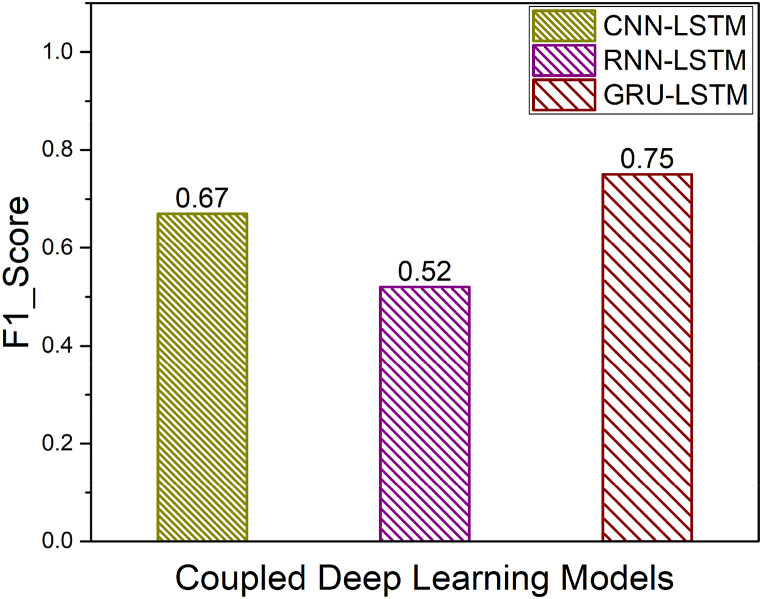
Comparison of F1_Scores of Coupled Models (CNN + LSTM vs. RNN + LSTM vs. GRU + LSTM).

**Fig. 16:**
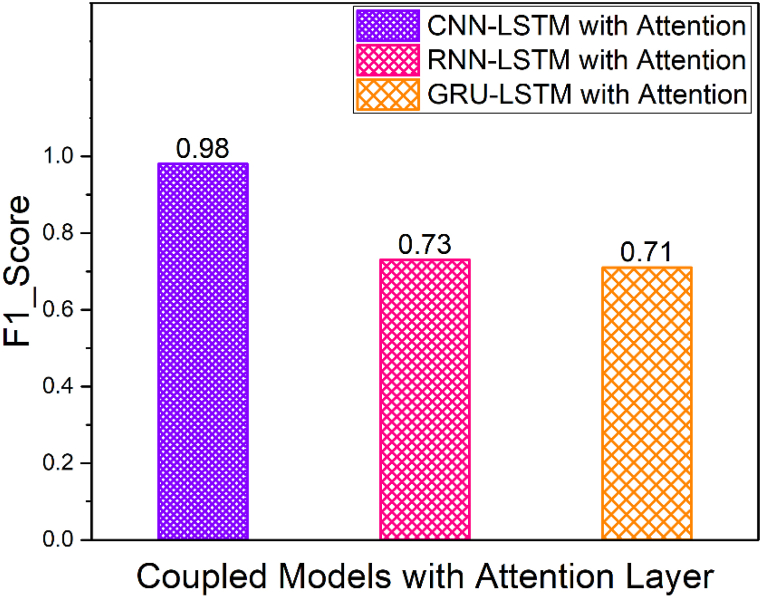
Comparison of F1_Scores of Coupled Models with Attention Layer (CNN + LSTM + ATT vs. RNN + LSTM + ATT vs. GRU + LSTM + ATT).

Fig. 14 shows the macro averaged F1_Score of simple models namely CNN, RNN, GRU and LSTM. Furthermore, the macro averaged F1_Score of Coupled Models namely CNN + LSTM, RNN + LSTM and GRU + LSTM is shown in Fig. 15. Also, the macro averaged F1_Score of Coupled Models with Attention layer namely CNN + LSTM + ATT, RNN + LSTM + ATT and GRU + LSTM + ATT is shown in Fig. 16. It is observed that the F1_Score of CNN-LSTM with attention layer is 0.98 which is higher compared to the other deep learning models utilized in this research work.

### 4.2 Development of a wearable device

The three different SoC devices namely Raspberry PI 3, Raspberry PI 4 and NVIDIA Jetson Nano developer kit were utilized in this work as an edge computing device. However, the Raspberry PI 3 is old, it is one of the most common and widely used board in India. Also, the Raspberry PI 3 Model B+ are available and can be purchased for a least price from local vendors through online mode. So, the same has been used in this study. The specification and performance metrics of all these three hardware boards are compared and the values are presented in Table 3.

**Table 3:** Specifications and metrics of proposed hardware device.

Parameters	Raspberry PI 3 Model B+	Raspberry PI 4 with 4 GB RAM	NVIDIA Jetson Nano developer kit with 4 GB RAM
Cost (in Rs.)	4949/-	5349/-	18,499/-
Size	Medium	Medium	Large
Availability	Production Stopped but Available	Available	Available
Power Consumption	12.5 Watts	15 Watts	5 Watts and 10 Watts mode
Computation time (Seconds)	9.446556	2.161639	0.43656

From Table 3 and it is observed that the cost (in Rs.), size, availability, power consumption and computation time (in seconds) of three different edge computing devices namely Raspberry PI 3, Raspberry PI 4 and Jetson Nano developer board were presented in Table 3. Furthermore, it is evident that the cost of the NVIDIA Jetson Nano developer kit is more than thrice of Raspberry PI 3, Raspberry PI 4 however, the Jetson Nano has less computation time and less power consumption. Also, it is seen that the Raspberry PI 4 Model B has computation time of 2.161639 s and there is a no enormous difference in the pricing of Raspberry PI 3 and Raspberry PI 4 controller. Nowadays, the Raspberry PI 4 controller is coming with 8 GB RAM in which the computation time shall be further reduced.

**Fig. 17:**
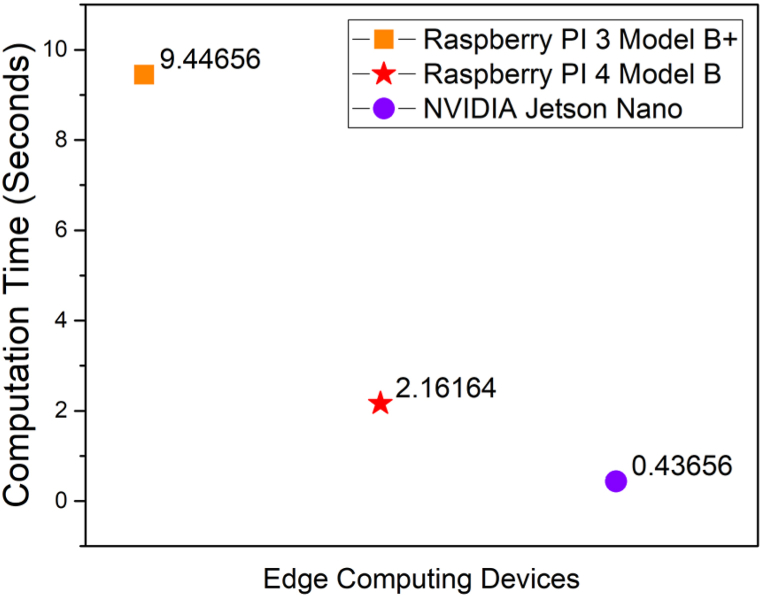
Comparison of computation time of different AI edge computing devices.

**Fig. 18:**
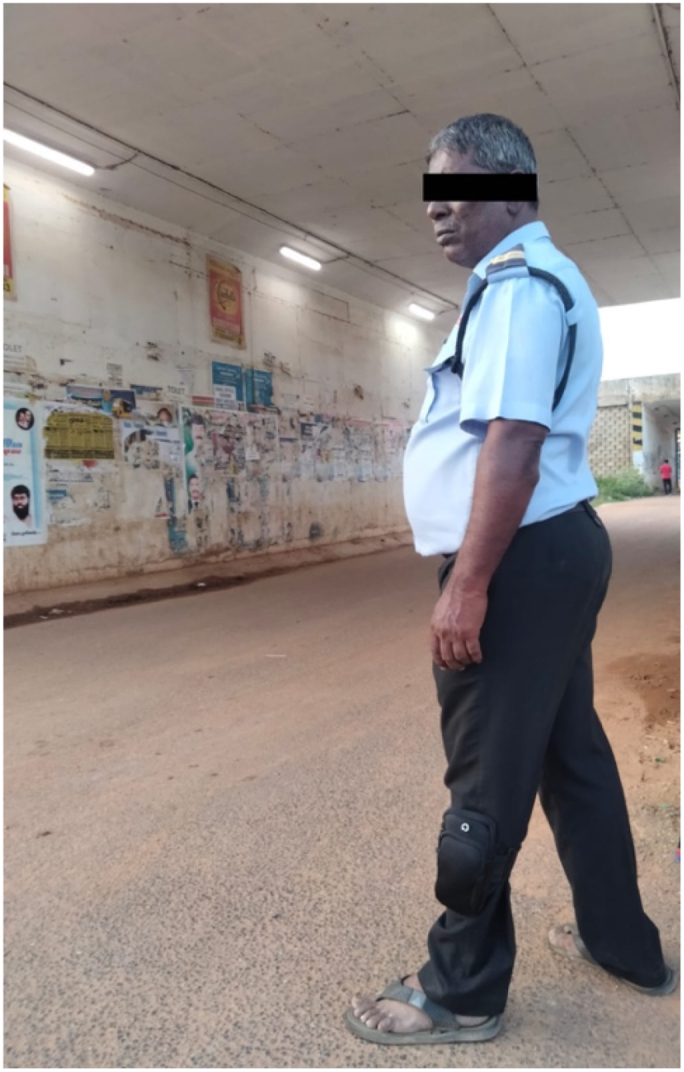
Proposed wearable device fixed to the elderly.

Fig. 17 shows the comparative analysis of computation time of three different AI edge computing devices. It is seen that the NVIDIA Jetson Nano developer kit has less computation time of 0.43656 s whereas the Raspberry PI 3 and Raspberry PI 4 have computation time of 9.44656 s and 2.16164 s respectively. So, the NVIDIA Jetson Nano based edge computing device can be used in the application where the money is not a concern whereas the performance of Raspberry PI 4 Model B is optimal and is better for elderly fall detection applications. Fig. 18 shows the proposed wearable device fixed to the elderly. It is observed that the wearable device is attached to the left leg of the elderly. Also, the proposed wearable device is attached to either right or left leg of the elderly.

**Fig. 19:**
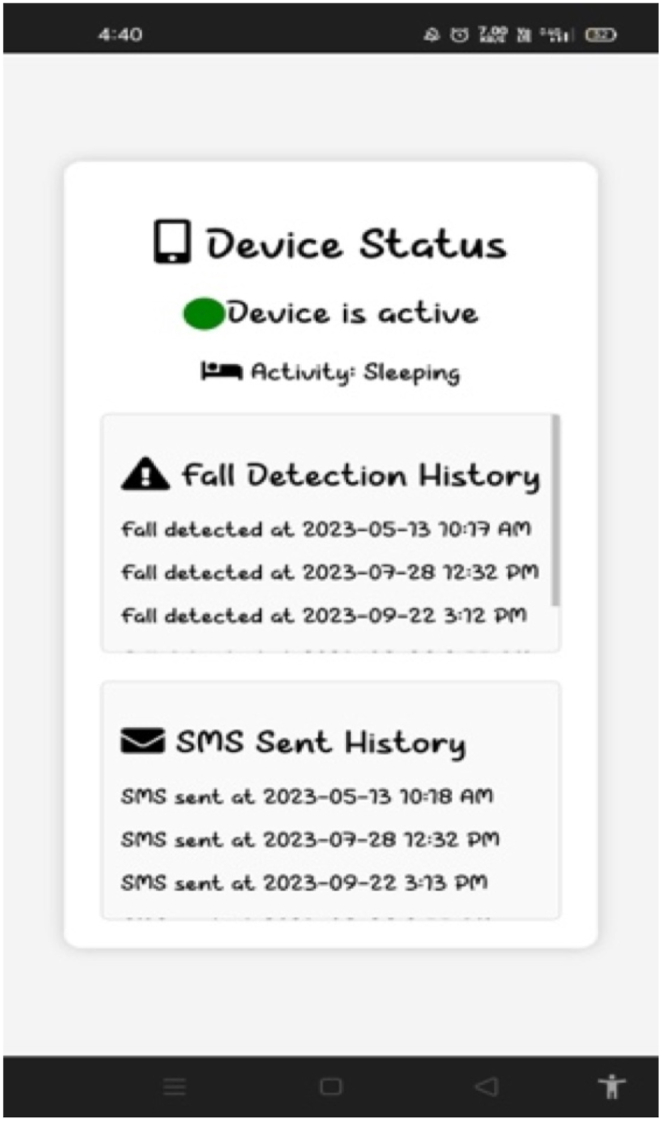
Gui for caretaker.

From Fig. 19, it is shown that the caretaker can visualize the device status, activity of the elderly, fall detection and SMS sent history remotely. In real-time, the AI enabled Edge Computing based Wearable device for Fall Detection and Prevention of Elderly People was developed using Raspberry PI 4. Furthermore, the accelerometer sensor is connected to the Raspberry PI 4 and the developed device was fixed to the elderly person using band. Also, the CNN-LSTM with attention model file (knowledge base) which was generated after the training phase were uploaded to the Raspberry PI 4 Model B. The pre-processing algorithm especially Finite Impulse Response (FIR) filter algorithm along with CNN-LSTM with attention were coded inside the Raspberry PI 4. Additionally, the Raspberry PI 4 is paired to internet with the help of in-built WiFi module. Once the device is paired, the wearable device records the ADL of the elderly people, feeds the quasi-live input to the pre-processing algorithm which filters the quasi-live stream data and stores it in.csv file which is later given to CNN-LSTM with attention algorithm. The CNN-LSTM with attention along with knowledge base predicts ADL and the ADL will be uploaded to the IoT cloud which can be monitored by caretaker using GUI is shown on Fig. 19. Further, it is observed that the device status, current activity and fall detection history can be monitored remotely by the caretaker using GUI. The developed edge computing device was meant to detect limited events such as fall, fall from bed, lying in bed, walking in uneven surface of elderly. Also, the maximum accuracy of the wearable device is limited to 97%. In near future, the various other events climbing/descending stairs, jogging, cycling etc. shall be utilized to train deep learning models increases versatility. Also, the recent Explainable AI (XAI) techniques shall be utilized to process the input data to enhance the accuracy of the deep learning models.

## 5 Limitations

The developed edge computing device was meant to detect limited events such as fall, fall from bed, lying in bed, walking in uneven surface of elderly and shall not detect intermediate events of elderly. The proposed framework utilizes stratified split for input data and the cross-validation is incorporated to mitigate potential bias. Also, the maximum accuracy of the wearable device is limited to 97%. In near future, the various other events climbing/descending stairs, jogging, cycling etc. shall be utilized to train deep learning models increases versatility. Also, the recent Explainable AI (XAI) techniques shall be utilized to process the input data to enhance the accuracy of the deep learning models.

## 6 Conclusion & future scope

Falls lead to death and serious injuries among elderly people every year. In this work, an AI edge computing based wearable device was developed to monitor fall detection and prevention of elderly. Also, the performance of various deep learning algorithms such as RNN, CNN, GRU, LSTM, CNN-LSTM, RNN-LSTM, GRU-LSTM, CNN-LSTM with attention, RNN-LSTM with attention and GRU-LSTM with attention were evaluated and compared. The GUI for caretakers was developed using HTML and the caretakers can monitor the activity of the elderly remotely. Results demonstrate that the performance metrics of CNN-LSTM model with attention layer is better when compared to other deep learning models. Also, the GRU-LSTM model with attention layer has accuracy, recall, precision, F1_Score, model training and testing time is 92%, 78%, 68%, 71%, 761.71 s and 4.85 s respectively. Furthermore, it is observed that the CNN-LSTM model with attention layer is implemented in three different edge computing devices namely Raspberry PI 3, Raspberry PI 4 and Jetson Nano device. And, it is demonstrated that the Raspberry PI 3 Model B is optimal since it is less expensive, compact and has computation time of 2.161639 s whereas Jetson Nano is highly expensive, has less power consumption and has computation time of 0.43656 s. Also, it is clearly experimented that the Raspberry PI 4 Model B is capable of notifying the caretaker in 2 s. In near future, the evolutionary optimizers can be incorporated in the utilized deep learning models and the accuracy shall be increased. Also, the neural stick based compact computing devices shall be used for commercialization aspects.

## Data availability statement

The datasets generated and analyzed in the current study are available from the corresponding author on reasonable request.

## Funding disclosure

This research received no external funding.

## Ethics declarations

This study was reviewed and approved by the Institutional Ethics Committee at Gleneagles Global Health City, with the approval number (BMHR/2023/0054).

## Informed consent statement

Informed consent was obtained from all subjects involved in the study.

## CRediT authorship contribution statement

**Paramasivam A:** Writing – original draft, Validation, Methodology, Conceptualization, Formal analysis, Investigation, Supervision, Writing – review & editing. **Ferlin Deva Shahila D:** Methodology, Conceptualization, Formal analysis, Investigation. **Jenath M:** Writing – review & editing, Methodology, Formal analysis, Investigation, Writing – original draft. **Sivakumaran T.S:** Methodology, Conceptualization, Formal analysis, Investigation, Validation, Writing – original draft. **Sakthivel Sankaran:** Investigation, Methodology, Writing – original draft, Writing – review & editing. **Pavan Sai Kiran Reddy Pittu:** Validation, Methodology, Data curation, Conceptualization, Investigation, Resources, Writing – original draft. **Vijayalakshmi S:** Writing – review & editing, Validation.

## Declaration of competing interest

The authors declare that they have no known competing financial interests or personal relationships that could have appeared to influence the work reported in this paper.
