# Supplementary material for: Development of artificial intelligence edge computing based wearable device for fall detection and prevention of elderly people
Source: Heliyon. 2024 Apr 9;10(8):e28688. doi: 10.1016/j.heliyon.2024.e28688 (PMC11019185; doi:10.1016/j.heliyon.2024.e28688)
Supplement: Multimedia component 1 [file mmc1.docx]

| CNN-LSTM with Atten | Output Shape | Parameter |
| --- | --- | --- |
|  |  |  |
| conv1d_91 (Conv1D) | (None, 254, 64) | 256 |
| max_pooling1d_91 (MaxPooling1D) | (None, 127, 64) | 0 |
| lstm_123 (LSTM) | (None, 127, 128) | 98816 |
| batch_normalization_234 (Batch Normalization) | (None, 127, 128) | 512 |
| flatten_91 (Flatten) | (None, 16256) | 0 |
| dense_194 (Dense) | (None, 64) | 1040448 |
| batch_normalization_235 (Batch Normalization) | (None, 64) | 256 |
| dense_195 (Dense) | (None, 5) | 325 |
| Dense Layer 6 (Dense):  Number of units/neurons: 64  Activation function: relu  Input dimension: 16256  Dense Layer 8 (Dense):  Number of units/neurons: 5  Activation function: softmax  Input dimension: 64 | | |

| CNN-LSTM | Output Shape | Parameter |
| --- | --- | --- |
|  |  |  |
| conv1d_34 (Conv1D) | (None, 254, 64) | 256 |
| max_pooling1d_34 (MaxPooling1D) | (None, 127, 64) | 0 |
| lstm_66 (LSTM) | (None, 127, 128) | 98816 |
| batch_normalization_120 (Batch Normalization) | (None, 127, 128) | 512 |
| flatten_34 (Flatten) | (None, 16256) | 0 |
| dense_80 (Dense) | (None, 64) | 1040448 |
| batch_normalization_121 (Batch Normalization) | (None, 64) | 256 |
| dense_81 (Dense) | (None, 5) | 325 |
| Dense Layer 6 (Dense):  Number of units/neurons: 64  Activation function: relu  Input dimension: 16256  Dense Layer 8 (Dense):  Number of units/neurons: 5  Activation function: softmax  Input dimension: 64 | | |

| CNN | Output Shape | Parameter |
| --- | --- | --- |
|  |  |  |
| Input_4 (InputLayer) | [(None, 256, 1)] | 0 |
| conv1d_3 (Conv1D) | (None, 254, 64) | 256 |
| max_pooling1d_3 (MaxPooling1D) | (None, 127, 64) | 0 |
| batch_normalization_6 (Batch Normalization) | (None, 127, 64) | 256 |
| flatten_3 (Flatten) | (None, 8128) | 0 |
| dense_6 (Dense) | (None, 64) | 520256 |
| batch_normalization_7 (Batch Normalization) | (None, 64) | 256 |
| dense_7 (Dense) | (None, 5) | 325 |
| Dense Layer 6 (Dense):  Number of units/neurons: 64  Activation function: relu  Input dimension: 8128  Dense Layer 8 (Dense):  Number of units/neurons: 5  Activation function: softmax  Input dimension: 64 | | |

| GRU-LSTM with Atten | Output Shape | Parameter | Connected to |
| --- | --- | --- | --- |
|  |  |  |  |
| input_83 (InputLayer) | [(None, 256, 1)] | 0 | [] |
| conv1d_155 (Conv1D) | (None, 254, 64) | 256 | ['input_83[0][0]'] |
| max_pooling1d_155 (MaxPooling1D) | (None, 127, 64) | 0 | ['conv1d_155[0][0]'] |
| gru_63 (GRU) | (None, 127,128) | 74496 | ['max_pooling1d_155[0][0]'] |
| lstm_187 (LSTM) | (None, 127,128) | 98816 | ['max_pooling1d_155[0][0]'] |
| concatenate_62 (Concatenate) | (None, 127,256) | 0 | ['gru_63[0][0]','lstm_187[0][0]'] |
| batch_normalization_360 (Batch Normalization) | (None, 127,256) | 1024 | ['concatenate_62[0][0]'] |
| flatten_154 (Flatten) | (None, 32512) | 0 | ['batch_normalization_360[0][0]'] |
| dense_320 (Dense) | (None, 64) | 2080832 | ['flatten_154[0][0]'] |
| batch_normalization_361 (Batch Normalization) | (None, 64) | 256 | ['dense_320[0][0]'] |
| dense_321 (Dense) | (None, 5) | 325 | ['batch_normalization_361[0][0]'] |
| Dense Layer 9 (Dense):  Number of units/neurons: 64  Activation function: relu  Input dimension: 32512  Dense Layer 11 (Dense):  Number of units/neurons: 5  Activation function: softmax  Input dimension: 64 | | | |

| GRU-LSTM | Output Shape | Parameter | Connected to |
| --- | --- | --- | --- |
|  |  |  |  |
| input_112 (InputLayer) | [(None, 256, 1)] | 0 | [] |
| conv1d_184 (Conv1D) | (None, 254, 64) | 256 | ['input_112[0][0]'] |
| max_pooling1d_184 (MaxPooling1D) | (None, 127, 64) | 0 | ['conv1d_184[0][0]'] |
| gru_92 (GRU) | (None, 127,128) | 74496 | ['max_pooling1d_184[0][0]'] |
| lstm_216 (LSTM) | (None, 127,128) | 98816 | ['max_pooling1d_184[0][0]'] |
| concatenate_91 (Concatenate) | (None, 127,256) | 0 | ['gru_92[0][0]','lstm_216[0][0]'] |
| batch_normalization_418 (Batch Normalization) | (None, 127,256) | 1024 | ['concatenate_91[0][0]'] |
| flatten_183 (Flatten) | (None, 32512) | 0 | ['batch_normalization_418[0][0]'] |
| dense_378 (Dense) | (None, 64) | 2080832 | ['flatten_183[0][0]'] |
| batch_normalization_419 (Batch Normalization) | (None, 64) | 256 | ['dense_378[0][0]'] |
| dense_379 (Dense) | (None, 5) | 325 | ['batch_normalization_419[0][0]'] |
| Dense Layer 9 (Dense):  Number of units/neurons: 64  Activation function: relu  Input dimension: 32512  Dense Layer 11 (Dense):  Number of units/neurons: 5  Activation function: softmax  Input dimension: 64 | | | |

| GRU | Output Shape | Parameter |
| --- | --- | --- |
|  |  |  |
| Input_8 (InputLayer) | [(None, 256, 1)] | 0 |
| gru_7 (GRU) | (None, 256, 128) | 50304 |
| batch_normalization_14 (Batch Normalization) | (None, 256, 128) | 512 |
| flatten_7 (Flatten) | (None, 32768) | 0 |
| dense_14 (Dense) | (None, 64) | 2097216 |
| batch_normalization_15 (Batch Normalization) | (None, 64) | 256 |
| dense_15 (Dense) | (None, 5) | 325 |
| Dense Layer 5 (Dense):  Number of units/neurons: 64  Activation function: relu  Input dimension: 32768  Dense Layer 7 (Dense):  Number of units/neurons: 5  Activation function: softmax  Input dimension: 64 | | |

| LSTM | Output Shape | Parameter |
| --- | --- | --- |
|  |  |  |
| input_3 (Input Layer) | [(None, 256, 1)] | 0 |
| lstm_2 (LSTM) | (None, 256, 128) | 66560 |
| batch_normalization_4 (Batch Normalization) | (None, 256, 128) | 512 |
| flatten_2 (Flatten) | (None, 32768) | 0 |
| dense_4 (Dense) | (None, 64) | 2097216 |
| batch_normalization_5 (Batch Normalization) | (None, 64) | 256 |
| dense_5 (Dense) | (None, 5) | 325 |
| Dense Layer 5 (Dense):  Number of units/neurons: 64  Activation function: relu  Input dimension: 32768  Dense Layer 7 (Dense):  Number of units/neurons: 5  Activation function: softmax  Input dimension: 64 | | |

| RNN-LSTM with Atten | Output Shape | Parameter | Connected to |
| --- | --- | --- | --- |
|  |  |  |  |
| input_8 (Input Layer) | [(None, 256, 1)] | 0 | [] |
| lstm_31 (LSTM) | (None, 256,128) | 66560 | ['input_8[0][0]'] |
| dot_7 (Dot) | (None, 256,256) | 0 | ['lstm_31[0][0]','lstm_31[0][0]'] |
| batch_normalization_54 (Batch Normalization) | (None, 256,256) | 1024 | ['dot_7[0][0]'] |
| simple_rnn_27 (SimpleRNN) | (None, 64) | 20544 | ['batch_normalization_54[0][0]'] |
| batch_normalization_55 (Batch Normalization) | (None, 64) | 256 | ['simple_rnn_27[0][0]'] |
| dense_27 (Dense) | (None, 5) | 325 | ['batch_normalization_55[0][0]'] |
| Dense Layer 7 (Dense):  Number of units/neurons: 5  Activation function: softmax  Input dimension: 32512 | | | |

| RNN-LSTM | Output Shape | Parameter |
| --- | --- | --- |
|  |  |  |
| lstm_11 (LSTM) | (None, 256, 128) | 66560 |
| batch_normalization_22 (Batch Normalization) | (None, 256, 128) | 512 |
| simple_rnn_11 (SimpleRNN) | (None, 64) | 12352 |
| batch_normalization_23 (Batch Normalization) | (None, 64) | 256 |
| dense_11 (Dense) | (None, 5) | 325 |
| Dense Layer 5 (Dense):  Number of units/neurons: 5  Activation function: softmax  Input dimension: 64 | | |

| RNN | Output Shape | Parameter |
| --- | --- | --- |
|  |  |  |
| input_137 (Input Layer) | [(None, 256, 1)] | 0 |
| conv1d_209 (Conv1D) | (None, 254, 64) | 256 |
| max_pooling1d_209 (MaxPooling1D) | (None, 127, 64) | 0 |
| simple_rnn_52 (SimpleRNN) | (None, 127, 128) | 24704 |
| batch_normalization_468 (Batch Normalization) | (None, 127, 128) | 512 |
| flatten_208 (Flatten) | (None, 16256) | 0 |
| dense_428 (Dense) | (None, 64) | 1040448 |
| batch_normalization_469 (Batch Normalization) | (None, 64) | 256 |
| dense_429 (Dense) | (None, 5) | 325 |
| Dense Layer 7 (Dense):  Number of units/neurons: 64  Activation function: relu  Input dimension: 16256  Dense Layer 9 (Dense):  Number of units/neurons: 5  Activation function: softmax  Input dimension: 64 | | |
